# Genetic Engineering of Zebrafish in Cancer Research

**DOI:** 10.3390/cancers12082168

**Published:** 2020-08-04

**Authors:** Ludivine Raby, Pamela Völkel, Xuefen Le Bourhis, Pierre-Olivier Angrand

**Affiliations:** Univ. Lille, CNRS, Inserm, CHU Lille, UMR9020-U1277–CANTHER–Cancer Heterogeneity Plasticity and Resistance to Therapies, F-59000 Lille, France; ludivine.raby.etu@univ-lille.fr (L.R.); pamela.voelkel@univ-lille.fr (P.V.); xuefen.le-bourhis@univ-lille.fr (X.L.B.)

**Keywords:** zebrafish, cancer model, chemical carcinogenesis, genetic screens, TILLING, genome editing, transgenesis

## Abstract

Zebrafish (*Danio rerio*) is an excellent model to study a wide diversity of human cancers. In this review, we provide an overview of the genetic and reverse genetic toolbox allowing the generation of zebrafish lines that develop tumors. The large spectrum of genetic tools enables the engineering of zebrafish lines harboring precise genetic alterations found in human patients, the generation of zebrafish carrying somatic or germline inheritable mutations or zebrafish showing conditional expression of the oncogenic mutations. Comparative transcriptomics demonstrate that many of the zebrafish tumors share molecular signatures similar to those found in human cancers. Thus, zebrafish cancer models provide a unique in vivo platform to investigate cancer initiation and progression at the molecular and cellular levels, to identify novel genes involved in tumorigenesis as well as to contemplate new therapeutic strategies.

## 1. Introduction

As early as 1902, Marianne Plehn at the Versuchsstation für Fischrei (Munich, Germany) pioneered the description of cancer in fish and recorded various cases of cancers in different salmonoids and cyprinoids [1]. Among early studies, in the 1920s and beginning of 1930s Curt Kosswig (Universität Münster, Germany), Georg Häussler (Institut für Experimentelle Krebsforschung, Heidelberg, Germany) and Myron Gordon (Cornell University, NY, USA) independently observed that interspecies hybrids between strains of the southern platyfish *Xiphophorus maculatus* and the green swordtail *Xiphophorus helleri* spontaneously develop malignant melanomas [2,3,4,5]. This groundbreaking observation conceptualizes the genetic basis and the heredity of certain cancers, about 40 years before the discovery of the first oncogenes. Indeed, these *Xiphophorus* neoplasms originate from the macromelanophores as the result of the interaction of two genetic traits. In southern platyfish, a tumor suppressor (*cdkn2x*) conceals the effects of an oncogene (*xmrk*). When green swordtails are mated by artificial insemination with the interspecies hybrids, the segregation of the tumor suppressor gene from the oncogene is responsible for melanoma formation according to Mendelian laws [6,7]. Over the years, spontaneous and induced neoplasms from a spectrum of tissues were observed in more than 200 fish species farmed or raised in aquaria and belonging to a large number of families including Poeciliidae (livebearers), Cyprinidae (carps and minnows), Cichlidae (cichlids), Cyprinodontidae (killifish), Characidae (tetras), Adrianichthyidae (medakas), Aplocheilidae (rivulins), Anguillidae (anguillas), Percidae (perches) or Salmonidae (salmons and trouts) [8]. These cancers originate from all tissues and are highly similar to human tumors at the histological and cellular levels raising the idea that fish could be used as cancer models and within the various fish models, zebrafish has progressively became central to cancer research.

The zebrafish (*Danio rerio*) is a small (3 to 4 cm long) freshwater fish belonging the Cyprinidae family that lives in rivers and rice paddies in India, Nepal and Bangladesh [9,10]. It emerged as a model to study early development of the embryo in the 1930s, but rapidly expanded into a larger area of research [11]. The success of the zebrafish in experimental biology is mainly due to its attributes that include: (i) the large number of progeny (100–200 embryos per clutch) offering high confidence in statistical analysis; (ii) the production of optically clear embryos that undergo rapid development ex utero; (iii) the manipulability of its embryology [12]; (iv) comparison of the zebrafish genome to the human reference genome shows that about 70% of human genes have at least one zebrafish orthologue (when considering disease-causing human genes the orthology is up to 80%) [13]; (v) the ability of zebrafish to absorb molecules that are dissolved in water allowing drug assays and screens [14,15]; and (vi) the reduced zebrafish husbandry expenses. In addition, transparent mutant lines like Casper and Crystal have been developed to facilitate in vivo imaging throughout all stages of zebrafish including adults [16,17]. Similarly, a plethora of transgenic zebrafish lines such as Tg(fli1:EGFP) [18] and Tg(flk1:EGFP) [19] having their entire vascular system labeled with green fluorescence or Tg(gata1:DsRed) [20] with red fluorescent blood cells allows to follow the development and location of different organs and cells in vivo.

This review aims to provide an overview of the genetic approaches making the zebrafish an excellent model in cancer research, as first proposed in 2002 by Leonard Zon and colleagues (Children’s Hospital, Boston, MA, USA) [21].

## 2. Chemical Carcinogenesis

Zebrafish spontaneously develop, although rarely, almost any type of tumors [22,23,24]. These tumors usually occur after the age of 1 or 2 years. Zebrafish was also the first fish species subjected to chemical exposure for cancer research. In the 1960s, Mearle Stanton (National Institute of Cancer, Bethesda, MD, USA) exposed zebrafish to the carcinogen diethylnitrosamine (N-nitroso-diethylamine, DEN), and observed that they developed hepatic neoplasias [25]. A number of chemical compounds known to be carcinogenic in mammals also induce tumor formation in zebrafish [26,27,28,29,30,31] (Table 1).

Systematic studies showed that N-nitrosodimethylamine (NDMA) induces only liver tumors [26] and N-nitrosodiethylamine (DEN) primarily induces liver and pancreas carcinomas [27], whereas 7,12-dimethylbenz[a]anthracene (DMBA) and N-methyl-N’-nitro-N-nitrosoguanidine (MNNG) can induce a broad tumor spectrum, including epithelial, mesenchymal and neural tumors [28,30]. In contrast, ethylnitrosourea (ENU) exposure is more selective, with most treated fish developing epidermal papillomas but no invasive skin cancers [31]. These fish also exhibit cavernous hemangiomas and malignant peripheral nerve sheath tumors at low incidence. Taken together, these data demonstrate the capacity of the zebrafish to develop a diverse range of cancers that pathologically resemble the human tumor types.

DMBA is a carcinogen metabolized in the liver and able to induce in zebrafish a spectrum of liver lesions corresponding to the different stages of neoplastic progression in human, from dysplastic nodules to high graded hepatocellular carcinomas. Using oligonucleotide microarrays, gene expression signatures of zebrafish liver tumors were compared to normal zebrafish liver and human liver cancers [29,32]. The study revealed a marked dysregulation in hallmark cancer genes including genes associated with cell cycle, cytoskeletal organization, metastasis, RNA processing and protein synthesis. Furthermore, the study shows that there are strong similarities at the transcriptomic level between zebrafish and human liver tumors that extend to tumor progression [29]. Thus, zebrafish neoplasms appear closely related to human cancers in terms of histopathology and in terms of alteration of gene expression programs.

Chemical carcinogenesis in zebrafish easily generates models that resemble to human cancers. However, the approach has limits linked to late tumorigenesis onset, low incidence of tumor development, or genetic and location heterogeneities of the induced tumors. Furthermore, zebrafish chemical carcinogenesis models cannot be propagated as lines. Thus, the full potential of the zebrafish model in cancer research would require the generation of fish lines harboring genetic alteration in cancer genes.

## 3. Forward Genetics and Zebrafish Mutagenesis Screens

**Figure 1:**
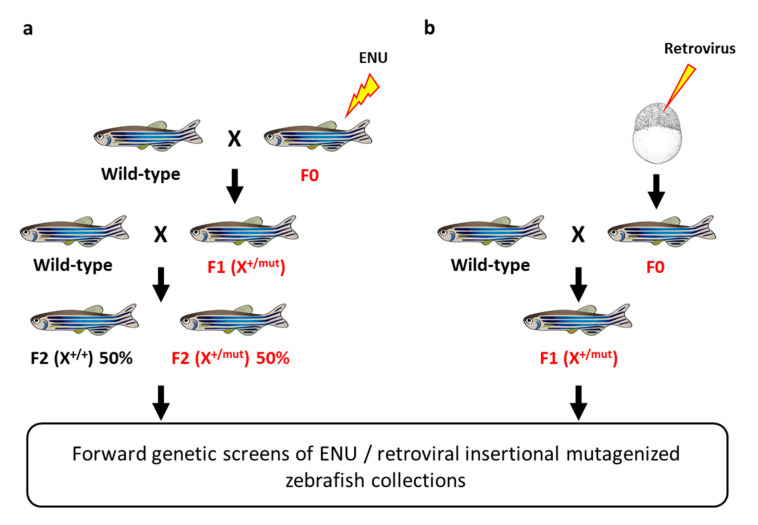
Strategies for forward genetic screens in zebrafish. (**a**) Chemical mutagenesis. Adult male zebrafish are treated with N-ethyl-N-nitrosourea (ENU) mutagen. Mutations induced in the germ cells can be propagated to the next generation through breeding wild-type females. Each F1 offspring contains a unique set of mutations; (**b**) Retroviral insertional mutagenesis. High-titer retroviruses are injected into 1K-stage embryos generating F0 mosaic zebrafish. Germ-line mutations are propagated in F1 through mating with wild-type fish.

N-ethyl-N-nitrosourea (ENU) is an alkylating agent preferentially responsible for A to T transversions and AT to GC transitions and therefore used as a potent point mutagen in *Drosophila* [33,34], mouse [35], as well as in zebrafish [12,36]. In the 1990s, two large-scale ENU-based mutagenesis screens in zebrafish were simultaneously conducted by Wolfgang Driever (Massachusetts General Hospital, Charlestown, MA, USA), Christiane Nüsslein-Volhard (Max-Planck-Institut für Entwicklungsbiologie, Tübingen, Germany) and their coworkers and led to the identification of thousands of mutants showing developmental defects [37,38] (Figure 1a). In addition to the enormous impact they have in the field of developmental genetics, such large-scale mutagenesis screens occasionally resulted in the identification of mutant zebrafish lines with an increased incidence of spontaneous neoplasia or a higher sensitivity chemical carcinogenesis (Table 2). For instance, in a forward genetic screen for mutations affecting ciliary motility in a large-scale ENU mutagenesis zebrafish library, Freek van Eeden and colleagues (Hubrecht Laboratory, Utrecht, The Netherlands) isolated a zebrafish mutant line carrying a mutation in the *dnaaf1* (*lrrc50*) gene [39]. While the homozygous mutants died during larval development due to severe ciliopathy phenotypes, heterozygous mutants develop to adulthood without apparent defects. However, a high seminoma prevalence is observed in the male population during the second and third year of life, with a penetrance exceeding 90% [40]. Subsequently, the authors showed that mutations in *DNAAF1* are also associated with human seminoma. Similarly, works from James Amatruda’s lab (University of Texas Southwestern Medical Center, Dallas, TX, USA) showed that nonsense mutations in the bone morphogenetic protein receptor gene *bmpr1bb* (*alk6b*) is responsible for testicular germ cell tumors [41]. These examples nicely demonstrate that forward genetic screens in zebrafish allows the identification of novel tumor suppressor genes involved in cancer.

The suitability of the zebrafish model in forward genetic screens was further demonstrated by the Nancy Hopkins lab (Massachusetts Institute of Technology, Cambridge, MA, USA) using retroviral insertional mutagenesis [42] (Figure 1b). Amsterdam and collaborators noticed that several heterozygous lines from this large-scale insertional mutagenesis displayed a high mortality rate, sometimes exceeding 50%, with the presence of tumors by the age of 2 years [43]. Out of the 12 heterozygous mutant zebrafish lines developing malignant peripheral nerve sheath tumors (MPNSTs), one of them harbored a mutation in the neurofibromatosis type 2 (*nf2a*) gene, an orthologue of the *NF2* tumor suppressor gene, while all the others carried retroviral insertions within ribosomal protein genes, linking protein biogenesis to cancer.

Retroviral insertion causes gene inactivation by disruption of coding or splicing sequences, but may also be responsible for gene activation due to the enhancer activity of the viral long terminal repeats (LTRs). In this respect, the retroviral insertional screen conducted by Nancy Hopkin and co-workers led to the discovery that retroviral insertions at the *fbxw4* locus are responsible for the overexpression of the *fgf8* neighboring gene that is in turn involved in neuroblastoma tumorigenesis [44].

Due to the late onset and low incidence of the disease, large-scale genetic screens for tumor development in adult zebrafish remains somehow laborious in terms of fish housing because a high number of fish should be hosted during large periods of time. For this reason, several laboratories set up various strategies to screen for cancer-related phenotypes in zebrafish embryo or larvae. The laboratory of Leonard Zon searched for mutations increasing cell proliferation by whole-mount immunohistochemistry. The authors looked for an increase in phosphohistone H3 (H3S10ph) a marker of M-phase cells, in haploid embryos at 36 hours post-fertilization (hpf) and identified the mutants *crash&burn* (*crb*) and *cease&desist* (*cds*) [45,46]. The *crb* mutant harbors a *mybl2* (*bmyb*) loss-of-function mutation responsible for defects in cell cycle progression and for genomic instability [45]. The *cds* mutant carries a mutation in the *separase* (*espl1*) gene coding for a protease cleaving the cohesin complex at the metaphase-anaphase transition and thus involved in the segregation of sister chromatids [46]. Both *mybl2* and *espl1* mutations slightly increase (2 to 2.5-fold, respectively) cancer incidence when zebrafish embryos are treated with MNNG [45,46].

Marco Koudijs and coworkers (Hubrecht Laboratory, Utrecht, The Netherlands) used a similar proliferation screen on a large-scale ENU-mutagenesis mutant library [47]. Whole-mount in situ hybridization on mutant zebrafish embryos at 40 hpf with a proliferating cell nuclear antigen (PCNA) probe was used to identify mutations that disrupt the genes *sufu* (suppressor of fused), *hhip* (hedgehog interacting protein) and *ptach1* (patched 1). These three genes code for repressors of Hedgehog signaling, a pathway which is activated in a number of solid cancers [48,49]. Following a different approach, the laboratory of Joseph Yost (University of Utah School of Medicine, Salt Lake City, UT, USA) described an embryonic screen aiming at the identification of mutants deficient in ionizing gamma irradiation-induced apoptosis and found a mutation I166T in the tumor suppressor Tp53 [50]. Adult zebrafish carrying this mutation are also predisposed to cancer development, predominantly sarcomas.

Other large-scale forward genetic screens conducted at later developmental stages, on zebrafish larvae, also permitted the identification of genes involved in tumorigenesis. Indeed, zebrafish larvae carrying a mutation in the *llgl2* gene develop epidermal cell tumors at 5 days post-fertilization (dpf) [51,52], whereas mutations in the smooth muscle myosin *myh11* gene causes epithelial invasion and cystic expansion in the posterior intestinal lumen of 5 dpf zebrafish larvae [53].

Most of the forward genetic screens aiming at discovering zebrafish cancer-related phenotypes were performed on mutagenized wild-type lines. However, in some cases mutagenesis was conducted on mutant or transgenic lines in order to take advantage of the characteristics of these lines. By outcrossing ENU treated wild-type zebrafish males with *golden* (*gol^−/−^*) mutant females, Keith Cheng and collaborators (Pennsylvania State College of Medicine, Hershey, PA, USA) screened for genomic instability and loss-of-heterozygosity of the golden pigmentation gene which is scored in large batches of embryos by the presence of unpigmented patches of the retinal pigment epithelium of the eye [54]. Twelve *genomic instability* (*gin1* to *gin12*) mutations were identified. Heterozygous adult from all gin lines develop various spontaneous tumors in skin, colon, kidney, liver, pancreas, ovary, testis, and neuronal tissues outlining the strong connection between genomic instability and cancer. Unfortunately, the genes carrying the *gin* mutations have not yet been cloned. To identify T-cell cancer phenotypes, the Nikolaus Trede Lab (University of Utah, Salt Lake City, UT, USA) ENU treated Tg(lck:EGFP) male transgenic zebrafish expressing the green fluorescent protein (GFP) in T-cells. Three mutant lines, *hulk* (*hlk*), *shrek* (*srk*) and *oscar the grouch* (*otg*) were identified by fluorescence microscopy for enlarged or extra-thymic GFP expression [55]. These three mutant lines develop T-cell acute lymphoblastic leukemia (T-ALL) malignancies that are phenotypically related to oncogene-induced leukemia. However, the responsible mutations have not been cloned yet, underlying the fact that the identification of cancer genes from ENU-mutagenesis zebrafish screen both requires important zebrafish housing structures and consequent efforts to identify the corresponding mutated genes by positional cloning in the zebrafish cancer lines.

In summary, large-scale genetic screens in zebrafish successfully resulted in the identification of lines displaying mutations in orthologues of mammalian tumor suppressor genes such as *NF2* or *TP53*, as well as in the discovery of a variety of genes that were not previously linked to cancer, including among others, *mybl2*, *dnnaf1* or multiple genes encoding ribosomal proteins (Table 2).

## 4. Reverse Genetics and TILLING

**Figure 2:**
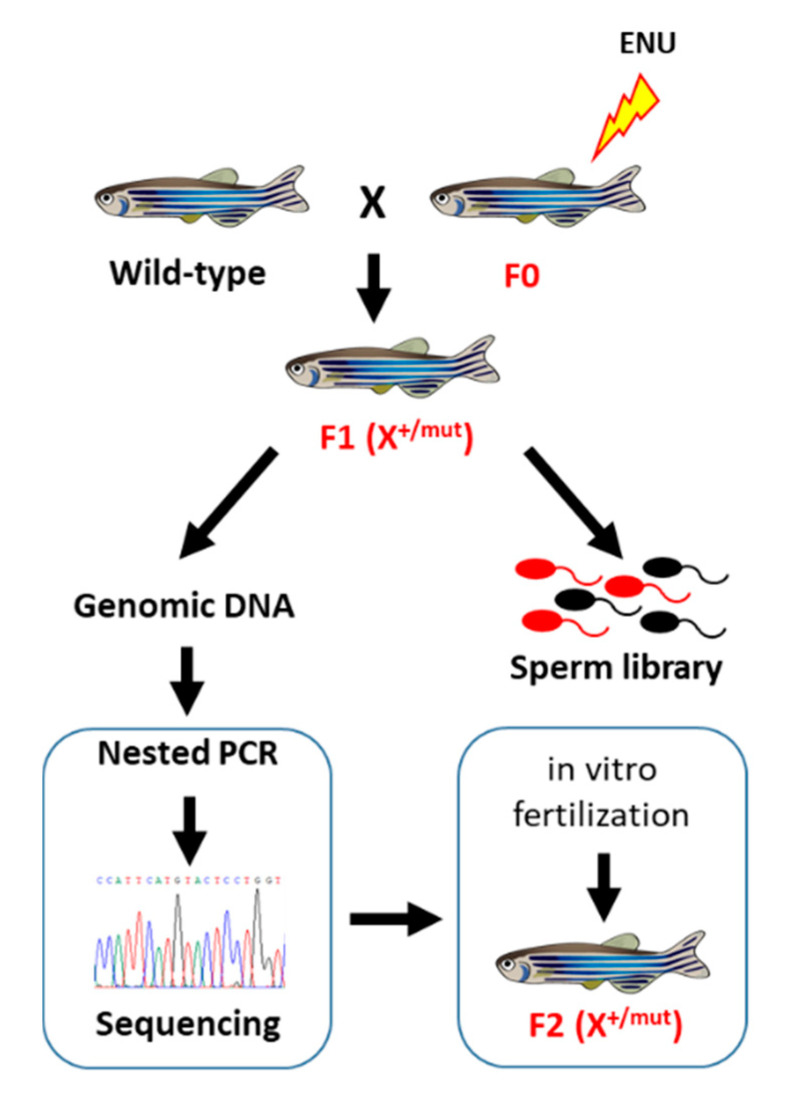
Overview of the target-selected mutagenesis in zebrafish. Male zebrafish treated with N-ethyl-N-nitrosourea (ENU) are mated to wild-type females. The resulting F1 male progeny are grown to breeding age and sacrificed to create a mutagenesis library consisting of genomic DNA, used to screen for F1 males harboring mutations in genes of interest (left) and cryopreserved sperm from each individual fish, for recovering the mutant lines using in vitro fertilization (right). This procedure permits the recovery of 50% heterozygous mutant zebrafish in the F2 generation.

In contrast to the unbiased nature of forward genetic screens that provide the opportunity to uncover novel genes involved in tumorigenesis, reverse genetic approaches allow the generation of zebrafish cancer models carrying mutations in known genes. Targeting Induced Local Lesions IN Genomes (TILLING) is a robust technology based on reverse genetics that may facilitate the identification of loss-of-function, hypomorphic or gain-of-function alleles in theoretically any selected gene. The method was first described in *Arabidopsis thaliana* in 2000 [56,57] but rapidly implemented in other organisms including mouse and zebrafish [58,59]. The approach consists in screening individual genomic DNA samples from a cohort of ENU-mutagenized F1 zebrafish to identify mutations that alter a chosen gene, while the sperm of the corresponding fish is cryopreserved for subsequent reconstitution of the mutant line by in vitro fertilization once desired mutations are identified (Figure 2). The screen for mutations could be done either using the CELI endonuclease cutting DNA heteroduplexes and thus identifying single base pair differences between mutant and wild-type alleles of target genes, and/or using DNA sequencing.

The laboratory of Ronald Plasterk (Hubrecht Laboratory, Utrecht, The Netherlands) was the first to apply the TILLING technology to zebrafish [59]. As a proof of concept, Plasterk and coworkers searched for mutations within the *rag1* gene, a gene involved in V(D)J recombination in T-cells, by sequencing the corresponding gene from 2679 F1 fish from a random ENU-mutagenized stock. They identified 15 mutations, including one that is responsible for the production of a truncated protein. The homozygous zebrafish line carrying this *rag1* loss-of-function mutation is deficient in V(D)J recombination but viable and fertile. Subsequently, this target-selected mutagenesis approach was used to generate several mutant lines for known tumor suppressor genes (Table 3).

The TILLING methodology has been used to generate a zebrafish line carrying a missense mutation in the DNA binding domain of Tp53 [60]. This mutation *tp53^M214K^* is similar to those found in a number of human cancers. The homozygous mutant embryos show a reduced gamma irradiation-induced apoptosis but develop normally. Interestingly, at the age of 8.5 months, 28% of *tp53^M214K^* mutant zebrafish develop malignant peripheral nerve sheath tumors (MPNST). Since *TP53* is the most frequently mutated tumor suppressor gene in human cancer, this mutant zebrafish line provides a powerful model to study the role of TP53 in carcinogenesis. The second most frequently mutated tumor suppressor in human cancers is *PTEN*. The study of *pten* in zebrafish is more complex since following a whole genome duplication that has occurred in the teleost genomes, zebrafish has retained two gene copies of *pten* in its genome, *ptena* and *ptenb*. However, mutations in both of these genes have been identified by TILLING [61]. The single homozygous mutants *ptena^−/−^*and *ptenb^−/−^*are viable with no developmental phenotype, whereas mutants lacking both *ptena* and *ptenb* (*ptena*^−/−^; *ptenb^−/−^*) die at day 5 dpf from pleiotropic defects. Interestingly, fish lacking three *pten* alleles (*ptena^+/−^*; *ptenb^−/−^*or *ptena^−/−^*; *ptenb^+/−^*) are viable and fertile but spontaneously develop ocular tumors diagnosed as hemangiosarcomas. This indicates that haploinsufficiency of the genes encoding Pten predisposes to cancer in zebrafish [62]. In humans, mutations in the tumor suppressor gene *APC* constitute the primary transforming event responsible for a large part of the sporadic and inherited colorectal cancers through the constitutive activation of the Wnt/β-catenin signaling pathway. A zebrafish *apc* loss-of-function mutation has been identified from an ENU mutagenesis library and homozygous *apc* mutant zebrafish die between 72 and 96 hpf with multiple defects including most prominently, cardiac malformations [66]. In contrast, the heterozygous *apc* mutant zebrafish do not present developmental defects but spontaneously develop intestinal adenomas, hepatomas and pancreatic adenomas [63]. The tumors accumulate β-catenin and express Wnt target genes showing that the signaling pathway is conserved and that *apc* mutant zebrafish line could serve as a model of familial adenomas polyposis. In humans, rare cases of young patients with brain tumors, skin neurofibromas, and café-au-lait spots that resemble the neurofibromatosis syndrome are associated with deficiencies in the mismatch repair (MMR) machinery genes *MSH2*, *MLH1*, *MSH6* or *PMS2* [67]. Using ENU-driven target-selected mutagenesis, zebrafish mutants with loss-of-function mutations in the *msh2*, *mlh1* and *msh6* genes have been described [64]. Homozygous mutant zebrafish in either one of these genes present genomic instability and develop tumors at an early age and low frequencies. These tumors are predominantly neurofibromas that mimic an important part of the phenotype of human patients, where biallelic MMR inactivation causes a neurofibromatosis type I-like phenotype. Inherited mutations in *BRCA2* predispose to breast and ovarian cancer. TILLING identified a nonsense mutation in *brac2* (*brca2^Q658X^*) at a gene position similar to *BRCA2* mutations found in humans with hereditary breast and ovarian cancers. Homozygous *brca2* mutant zebrafish are viable but fail to develop ovaries and develop into infertile males with deficient spermatogenesis [65]. Furthermore, *brca2* mutant zebrafish are predisposed to testicular neoplasia, while tumorigenesis is enhanced in a *tp53* deficient genetic background [65,68]. Thus, the *brca2* mutant zebrafish line shed new light on the role of *brca2* in ovary development and tumorigenesis in reproductive organs, as well as in cancer development associated to heritable *BRCA2* and *TP53* mutations.

Altogether, TILLING is a powerful technology that raise zebrafish as a pertinent model in cancer research. However, the approach suffers from several drawbacks: (i) The procedure is costly, labor intensive and cannot be implemented in most individual labs. (ii) The approach relies on random chemical mutagenesis. It might statistically be more difficult to obtain mutants for small-size genes and some sequences could have a weak mutagenic potential. (iii) The use of random chemical mutagenesis makes that in addition to the mutation of interest, several other mutations are present in the fish genome. ENU-induced mutants should be outcrossed for several generations in order to exclude effects of unknown additional mutations.

## 5. Gene Editing Technologies

**Figure 3:**
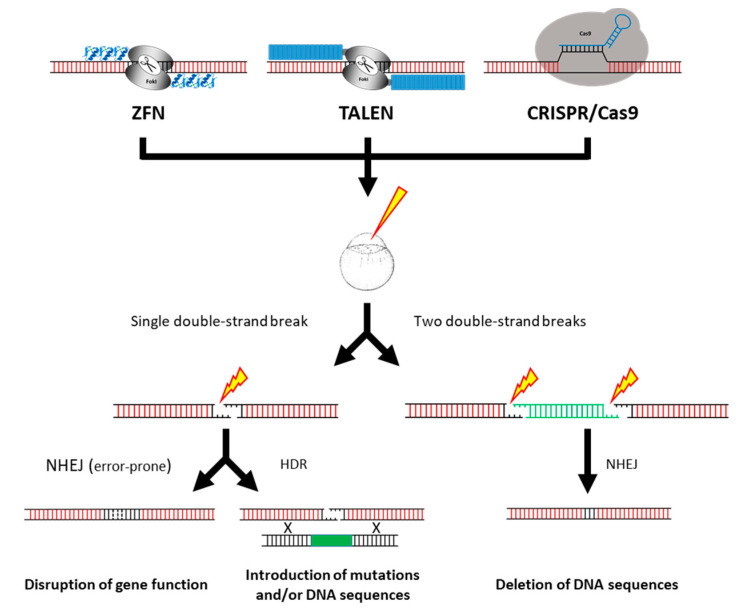
Gene targeting using programmable site-specific endonucleases. RNAs and/or proteins corresponding to the components of the ZFNs, TALENs and CRISPR/Cas9 programmable site-specific endonuclease systems are injected into zebrafish embryos at the 1-cell stage to induce double-strand DNA breaks at the target genomic sites. Cellular DNA repair mechanisms including non-homologous end joining (NHEJ) and homology-directed repair (HDR) are responsible for gene editing events such as the disruption of gene function, gene deletion or the introduction of precise modifications.

The discovery of the programmable site-specific endonucleases, as the most versatile and efficient tools to modify any genomic sequence, completely revolutionized the field of reverse genetics applied to the zebrafish disease model [69,70]. Programmable site-specific endonucleases are engineered to induce double-strand DNA breaks specifically at chosen genomic target sites. These double-strand DNA breaks subsequently stimulate cellular DNA repair mechanisms such as the error-prone non-homologous end joining (NHEJ) and the homology-directed repair (HDR). The NHEJ mechanism, which often results in small random nucleotide insertions or deletion (*indel*) at the cut site, can be used to disrupt the function of a gene. In contrast, HDR allows the insertion of precise genomic modifications in presence of a designed homologous DNA template. Another application of programmable site-specific endonucleases is the generation of large deletions, where two distant double-strand DNA breaks are induced on the same genomic DNA molecule (Figure 3). The programmable site-specific endonucleases used for genome editing are the zinc-finger nucleases (ZFNs), the transcription activator-like effector nucleases (TALENs) and the clustered regularly interspaced short palindromic repeats (CRISPR) RNA-guided Cas9 nucleases (CRISPR/Cas9) [71].

### 5.1. Zinc Finger Nucleases

Zinc finger nucleases (ZFNs) are chimeric proteins comprising a DNA binding domain composed of C_2_H_2_ type zinc fingers mainly derived from the transcriptional factor ZIF268/EGR1, and a cleavage domain from the bacterial endonuclease FokI [72]. The alpha helix of each zinc finger recognizes and binds to a 3 bp DNA sequence. Since the DNA binding domain of ZFNs is usually an array of three or four zinc fingers, it would recognize a nine to 18 bp target DNA sequence. Because FokI acts as a dimer, ZFNs function in pairs and cleave a spacer sequence to induce a double-strand DNA break located between the two ZFN binding sites. ZFNs were the first available direct gene targeting method in zebrafish, thus making any zebrafish gene knockout possible [73,74,75]. However, the engineering and selection of ZFNs with efficient and specific DNA binding activity remains highly challenging. In particular, the fact that the binding specificities of individual zinc fingers could overlap and depend on the context of the surrounding zinc fingers and DNA sequences renders their assembly difficult and has strongly limited the ZFN usages.

In spite of the difficulties associated to the design of efficient and specific ZFNs, the Thomas Look laboratory (Children’s Hospital, Harvard Medical School, Boston, MA, USA) successfully applied these programmable site-specific endonucleases to the inactivation of several cancer genes in zebrafish [76,77] (Table 4).

As for *PTEN*, the zebrafish genome codes for two orthologues of *NF1*, *nf1a* and *nf1b*. ZFN-mediated inactivation of these genes revealed that homozygous loss-of-function of *nf1a* or *nf1b* alone leads to phenotypically normal zebrafish. In contrast, loss of both genes results in larval lethality with phenotypes that resemble aspects of the human neurofibromatosis type 1 [76]. Moreover, Nf1 cooperate with Tp53 to the development of high-grade gliomas and MPNSTs in adult zebrafish. Indeed, the combined loss of *tp53* and three of four *nf1* alleles (*nf1a^+/–^*; *nf1b^–/–^*; *tp53^–/–^*) significantly accelerates the onset and increases the penetrance of tumors as compared with the loss of *tp53* alone (*nf1a^+/+^*; *nf1b^+/+^*; *tp53^–/–^*) or with the concomitant loss of *tp53* and both alleles of *nf1b* with intact *nf1a* (*nf1a^+/+^*; *nf1b^–/–^*; *tp53^–/–^*). On the other side, ZFN-inactivation of *tet2*, a gene coding for one of the TET family members of DNA methylcytosine oxidases that converts 5-methylcytosine into 5-hydroxymethylcitosine during the DNA demethylation process, is responsible for generalized myelodysplastic syndromes (MDS) in zebrafish [77]. The *tet2^−/−^*zebrafish mutants are viable and constitute a good model of MDS since somatic loss-of-function mutations of *TET2* are frequently found in human MDS.

### 5.2. Transcription Activator-Like Effector Nucleases

Transcription activator-like effector nucleases (TALENs) are similar to ZFNs in their architectures. Both programmable site-specific endonucleases contain the endonuclease domain of FokI, but differ in their DNA-binding domain moiety. The DNA binding domain of the TALENs derives from transcription activator-like effectors (TALEs) which are transcription factors from phytopathogens of the *Xanthomonas* genus. The DNA binding domain of TALE proteins is composed of an array of several 34 amino acids repeat units, with each repeat recognizing and binding to a single nucleotide in the target DNA sequence [91,92]. Within these 34 amino acids conserved modules, two residues at position 12 and 13, called RDV (repeat variable di-residue) determine the binding specificity to its target nucleotide. For instance, in the natural TALEs, the most frequent RDVs, NI (Asn Ile), NG (Asn Gly), HD (His Asp) and NN (Asn Asn)/NK (Asn Lys) specifically recognize the nucleotides A, T, C and G, respectively. Thus, compared to ZFNs, the DNA recognition basis of TALENs is much simpler and predictable, making the TALENs an extremely efficient programmable site-specific endonuclease system. With 15–17 repeated modules, the TALEN technology offers a way to introduce double-strand DNA breaks with a high specificity and a strong efficacy in any gene and any organism including zebrafish [93,94].

TALENs have been shown to be efficient enough to induce somatic mosaic bi-allelic mutations in tumor suppressor genes in F0 fish allowing the study of their function in tumorigenesis, even when homozygous mutants are larval lethal [78,79] (Table 4). TALEN-mediated somatic bi-allelic inactivation of *rb1* induces medulloblastoma-like primitive neuroectodermal tumors in wild-type zebrafish [79,80], whereas *cdkn2a/b* inactivation induces MPNST in the brain with high frequencies and early onset in F0 *tp53*-mutant zebrafish [79]. Using two pairs of TALENs designed to target both sides of the *tp53* gene (Figure 3), David Langenau and co-workers (Massachusetts General Hospital Research Institute, Boston, MA, USA) generate a *tp53^del/del^* zebrafish line [81]. Homozygous *tp53^del/del^* mutants start to develop tumors at 4 months of age, mainly leukemia with blast-like cells in the kidney marrow. At 7 months, angiosarcoma, MPNSTs or germ cell tumors are externally visible in a subset of *tp53^del/del^* zebrafish. This tumor spectrum is wider than what was observed for *tp53^M214K^* mutants identified by TILILNG and *tp53^I166T^* identified in an ENU forward genetic screen [50,60], indicating that tumor onset and phenotype might depend on the nature of the *tp53* mutation.

### 5.3. Clustered Regularly Interspaced Short Palindromic Repeats RNA-Guided Cas9 Nucleases

Cas9 is a dual RNA-guide DNA endonuclease associated with the clustered regularly interspaced short palindromic repeats (CRISPRs) playing a role of an adaptive immune system in archea and bacteria. In brief, a guide RNA (gRNA) recognizes a specific DNA sequence by hybridization and targets the Cas9 endonuclease that induces a double-strand DNA break at the RNA recognition site [95]. In contrast to ZFNs and TALENs that target DNA through a protein-DNA interaction, the recognition of the CRISPR/Cas9 target site is achieved via a RNA-DNA hybridization. In addition, while ZNFs and TALENs act as dimers, Cas9 induces double-strand DNA breaks as a monomer. The CRISPR/Cas9 can be programmed to cut different target sites by changing 20 nucleotides within the gRNA. The only limitation in designing a target site is the requirement for a protospacer adjacent motif (PAM) (NGG in the case of the *Streptococcus pyogenes* spCas9 enzyme) just adjacent to the 20-nt target sequence. Due to its remarkable simplicity, the CRISPR/Cas9 system rapidly became a genome-editing tool of choice in a wide variety of experimental model organism, including zebrafish [96]. The first use of CRIPSR/Cas9-mediated mutagenesis in zebrafish was demonstrated by Woong Hwang and colleagues (Massachusetts General Hospital, Charlestown, MA, USA) in 2013 [97]. The authors reported the targeting of 10 loci and showed mutagenesis rates between 24% and 59% in somatic tissues. At around the same time, Nannan Chang et al. (Pekin University, Beijing, China) showed that the CRIPSR/Cas9 is able to induce somatic bi-allelic conversion [98], while Li-En Jao et al. (Vanderbilt University School of Medicine, Nashville, TN, USA) demonstrated that CRISPR/Cas9-induced mutations are heritable, and the possibility to target five genomic loci simultaneously, resulting in multiple loss-of-function phenotypes in the same injected zebrafish [99]. Furthermore, the possibility to express the Cas9 protein under the control of tissue-specific promoters in transgenics renders tissue-specific gene disruption feasible in zebrafish [100,101].

While the CRISPR/Cas9 system appears to be an efficient and simple tool for genomic engineering, there are still several concerns over its specificity and possible unintended off-targets [102]. In zebrafish a study detected CRISPR/Cas9-mediated off-target mutagenesis in only 1/25 predicted off-target sites in germline, whereas in another study the off-target mutation rate was estimated between 1.1 to 2.5% [103,104]. These potential off-targets may easily be outcrossed away. Moreover, different Cas9 variants, including Cas9-HF1, eSpCas9, evoCas9 and HypaCas9, have been developed or under development to increase the specificity of the Cas9 enzyme and thus reduce the off-targets [105].

Despite a recent implementation, the CRISPR/Cas9 system has been already applied to the zebrafish cancer research field [82,83,84,85,86,87,88,89,90] (Table 4). Iroquois homeobox 1 (IRX1) is an homeobox-containing transcription factor playing a role in embryonic development and suspected to act as a tumor suppressor gene in head and neck squamous cell carcinoma, gastric cancers and glioma [106,107,108,109]. In zebrafish, Irx1 is encoded by two ohnologues *irx1a* and *irx1b*. The group of Seung Woo Park (Yonsei University College of Medicine, Seoul, Korea) used the TALEN technology to inactivate *irx1a*, while *irx1b* mutants were generated using the CRISPR/Cas9 system [82]. Homozygous mutants for both *irx1a* and *irx1b* (*irx1a^–/–^; irx1b^–/–^*) are malformed and die within 6 months of age. In contrast, single homozygous mutants for *irx1a* (*irx1a^–/–^*) and *irx1b* (*irx1b^–/–^*) are viable and fertile but spontaneously develop hyperplasia and tumors in different organs where the genes are expressed, including intestine, testis, ovary, kidney and bile duct. In a search for genetic alterations in mucosal melanomas, *SPRED1*, a negative regulator of MAPK signaling, was found inactivated in 37% of the tumors [83]. Through the expression of Cas9 under the control of the *mitfa* promoter in F0 mosaic zebrafish, Ablain et al. [83] showed that the melanocyte-specific targeting of *spred1* in addition to *ptena/b* and *tp53* results in late-onset melanomas, which is not observed through *ptena/b* and *tp53* targeting alone. This CRISPR/Cas9-mediated mosaic tissue-specific gene knockout could be combined to the concomitant expression of oncogenes in the F0 zebrafish through the co-injection of vectors driving the expression of Cas9, gRNAs and oncogenes. For instance, it has been shown that *NRAS^Q61R^* expression combined with *tp53* loss in melanocytes is responsible for tumor formation within 3 week after plasmid injection in the embryos. In contrast, *BRAF^V600E^* expression together with *cdkn2a* loss-of-function leads to melanoma after several months [83]. Similarly, using the *rag2* promoter to drive the expression of Cas9 and the Notch1a^ICD^ oncogene into immature T-cell progenitors, the Alejandro Gutierrez group (Boston Children’s Hospital, Boston, MA, USA) showed that inactivation of *ptch1* accelerates the Notch1a^ICD^-induced T-ALL onset [84]. These examples illustrate the usefulness of the CRISPR/Cas9 system to assay for tumor suppressor gene activities. On the other side, the approach may also be used to characterize genes promoting tumorigenesis. Using an RNAseq data set from human papillary thyroid cancers, Viviana Anelli et al. (New York Presbyterian Hospital, New York City, NY, USA) [90], identify TWIST2 as a key effector upregulated by the oncogenic BRAF^V600E^ mutation. The authors showed that the CRIPR/Cas9-inactivation of *twist3*, an orthologue of *TWIST2*, in a zebrafish *BRAF^V600E^* transgenic model, rescues defects in follicle structure and thyroid hormone production. These data indicate that *twist3* contributes to *BRAF^V600E^*-mediated transformation [90].

In sum, programmable site-specific endonucleases have been successfully used in zebrafish (i) to generate models harboring mutations in tumor suppressor genes (e.g., *nf1*, *rb1*; [76,78]); (ii) to engineer novel genetic alterations in tumor suppressor genes (e.g., *tp53^del/del^*; [81]); (iii) to demonstrate the tumor suppressor function of novel candidate genes (e.g., *irx1*, *spred1*, *arid1a* [82,83,87]); (iv) to characterize the role of genes involved in tumor development (e.g., *twist3*; [90]); (v) as well as to investigate the cooperation between different mutations in the tumorigenesis onset (e.g., *tp53* and *nf1* with *atrx* or *suz12* [85,86]). The use of programmable site-specific endonucleases can be applied to the engineering of zebrafish mutant lines [76,77], but also to the rapid generation of F0 mosaic somatic mutants [78,79,83,84,90]. In addition, expression of Cas9 under the control of tissue-specific promoters allows CRISPR/Cas9-mediated inactivation of cancer genes in specific tissues [83,84]. Finally, the programmable site-specific endonucleases have been mainly used to induce *indel* mutations and in some cases, used to generate genomic deletions via the targeting of two double-strand breaks [81,88] (Figure 3). However, to our knowledge, precise point mutations and knock-ins generated through HDR have not yet been reported in zebrafish in the context of cancer research.

## 6. Transgenesis

**Figure 4:**
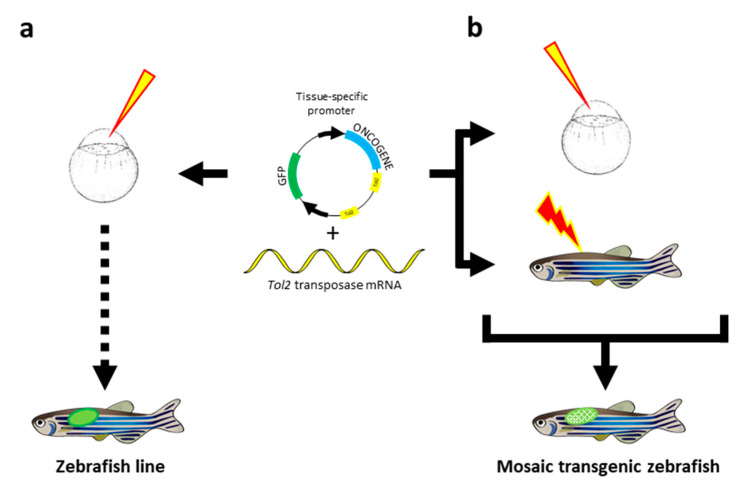
Tol2-mediated transgenesis in zebrafish. (**a**) Stable transgenesis. Transposase mRNA synthesized by in vitro transcription and a plasmid DNA harboring the *Tol2* cis regulatory elements (yellow) are co-injected into zebrafish fertilized eggs. The transposase synthesized from mRNA catalyzes the integration of the excised transgenic sequences placed between the *Tol2* short terminal repeats into the genome. The injected embryos are raised and crossed with wild-type fish. The integrated transgene is transmitted to the F1 generation showing non-mosaic transgene expression. (**b**) Mosaic F0 transgenic zebrafish can be created either through the microinjection into fertilized eggs or through electroporation in adults (TEAZ) of the Tol2 transposase and the construct containing the cis regulatory elements. A fluorescent marker gene (e.g., GFP) is often present in the construct to identify the transgenics.

The ability to generate transgenic lines that stably express genes (e.g., genes involved in diseases or fluorescent marker genes) is central to numerous biomedical studies using zebrafish as a model organism. The Monte Westerfield lab (University of Oregon, Eugene, OR, USA) pioneered the stable introduction of exogenous DNA into the zebrafish genome in the late 1980s [110]. After injection of a linearized plasmid into the cytoplasm of fertilized eggs, these first transgenic zebrafish transmitted the bacterial DNA to their progeny with a germline transmission rate of about 15%. However, gene expression from the transgenic cassettes was often subjected to transcriptional silencing, presumably as the result of methylation and rearrangements of the foreign DNA integrated as concatemers [111,112,113]. This limitation was bypassed by flanking the transgenic cassettes with 18-bp recognition sites for the *Saccharomyces cerevisiae* I-SceI meganuclease enzyme. Co-injection into the embryo of the enzyme together with the transgenic vector carrying the restriction sites, linearizes the circular DNA before genome integration, reduces concatemerization, and favors transgene expression. Although this strategy was first implemented in the Japanese rice fish medaka (*Oryzias latipes*) by the group of Jean-Stéphane Joly (Institut de Neurobiologie A. Fessard, Gif-Sur-Yvette, France) [114], the approach became widely applied to the generation of transgenic zebrafish lines [115]. An alternative method based on the *Tol2* transposon identified in the medaka fish has been developed by Koichi Kawakami (The Graduate University of Advanced Studies, Mishima, Shizuoka, Japan) [116,117,118]. *Tol2* is an active transposon element that belongs to the hAT transposon family. The principle of its use in transgenesis is based on the separation of the cis transposable elements called the short terminal repeats from the transposase enzyme. The transposase is in vitro transcribed and co-injected with a circular plasmid carrying the transgenic cassette flanked by the cis regulatory short terminal repeats (Figure 4a). This system allows the insertion of up to 11 kbp sequences of foreign DNA into the genome and produces germ-line transmission of the transgene in up to 50% of the injected zebrafish [119]. Due to transposon-mediated insertion, the prokaryotic sequences from the plasmid backbone are not integrated in the zebrafish host genome and transgenesis results in the integration of single copy per locus avoiding concatemer formation. The absence of concatemer integration together with the exclusion of the prokaryotic sequences prevents transgene silencing through heterochromatinization or chromosomal rearrangements. Finally, *Tol2*-mediated transgenesis usually leads to single copy insertions into the genome. All of these attributes made the *Tol2*-based approach a powerful and widely used methodology to generate transgenic zebrafish [120].

In 2003, in a landmark publication, David Langenau and coworkers (Harvard Medical School, Boston, MA, USA) were the first to demonstrate transgenic modeling of cancer in zebrafish [121]. The expression of murine Myc oncogene fused to GFP was targeted to the developing lymphocytes using the zebrafish *rag2* promoter. The transgenics developed T-ALL between 1 month and 5 months of age and the GFP-labeled leukemic cells were shown spreading from the thymus into the surrounding tissues and invading skeletal muscle and visceral organs. This study demonstrated that the expression of an oncogene is able to drive cancer formation in zebrafish. Furthermore, the co-expression of fluorescent markers allows in vivo monitoring of tumor progression in real-time, tumor cell isolation for further phenotypic or transcriptomic characterization as well as for serial transplantation into irradiated recipient zebrafish. These observations substantiate the power of the zebrafish model in cancer research and have opened avenues to the development of zebrafish transgenic lines applied to cancer modeling. Thus, over the years a large number of zebrafish lines expressing various oncogenes have been generated (Table A1).

**Figure 5:**
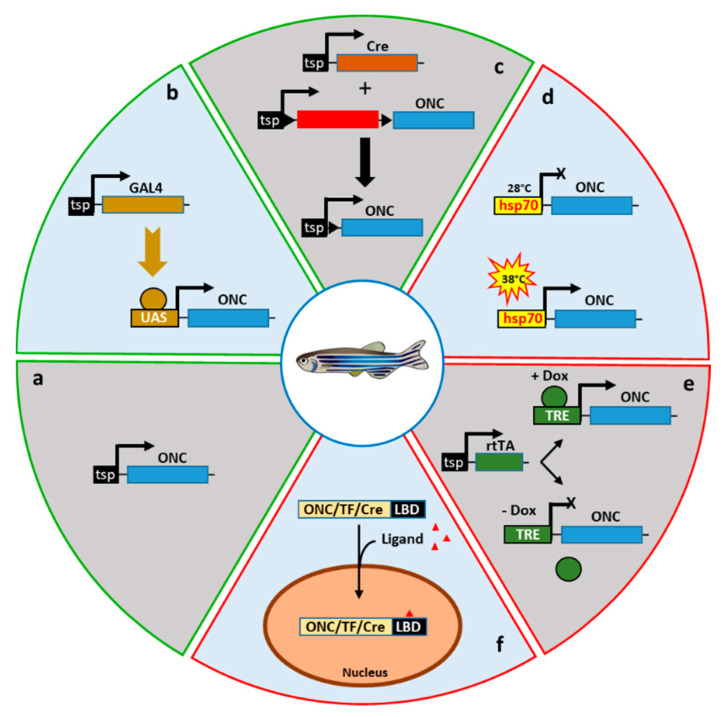
Strategies to control oncogene expression in zebrafish cancer models. (**a**) Spatial regulation of oncogene (ONC) expression through the activity of tissue-specific promoter (tsp). (**b**) The Gal4/ UAS system to enable cell type specific oncogene expression. The yeast transcription factor Gal4 is expressed through the control of a tissue-specific promoter (tsp) and binds to the upstream activating sequence (UAS) to drive the expression of the oncogene (ONC). (**c**) Cre-mediated oncogene (ONC) expression. Cre-mediated recombination removes an intervening sequence and places the oncogene under the control of a promoter. (**d**) Temporal oncogene (ONC) activation through the induction of the activity of the heat shock promoter. (**e**) Temporal oncogene (ONC) activation using the Tet-ON system. The binding of doxycycline to rtTA allows the transcription factor to bind and activate transcription at tetO responsive elements (TRE)-containing promoters. (**f**) Ligand-dependent oncogene (ONC) activation. Fusing the ligand-binding domain (LBD) onto oncogenic transcription factors (MYC), transcription factors (LexA) or Cre recombinase confers ligand-dependent activities to these proteins. Strategies to spatially control oncogene expression are boxed in green, whereas temporal to control of oncogene expression is boxed in red.

However, transgenic zebrafish expressing oncogenes could sometime not survive sexual maturity. The transgenic Tg(rag2:GFP-Myc) zebrafish line for instance, shows a 100% leukemia incidence with a cancer onset mostly preceding reproductive age. Consequently, maintenance and propagation of the line requires labor-intensive in vitro fertilization [122]. Thus, the full exploitation of the transgenic zebrafish model in cancer research necessitates the development of strategies to allow conditional expression of the oncogenes in a spatial- and temporal-controlled fashion. Moreover, cancer phenotypes mostly result from somatic mutations rather than from germline genetic mutations. Then, controlled activation of oncogenes in zebrafish somatic tissues would also accurately mimic cancer onset. Fortunately, an exceptional toolbox of conditional transgenic approaches mainly adapted from *Drosophila* and mouse genetics, has been established in zebrafish (Figure 5).

### 6.1. Tissue-Specific Promoters

In zebrafish, a number of tissue-specific promoters have been reported to faithfully reproduce endogenous gene expression patterns in transgenics. These tissue-specific promoters may be used to drive oncogene expression in define tissues (Figure 5a). In their princeps experiments, Langenau and colleagues [121] used the *rag2* promoter to drive expression of the *Myc* oncogene and induce T-ALL. However, since B-lymphoblasts also express *rag2* and because *MYC* is known to drive human B-ALL, the Tg(rag2:GFP-Myc) and Tg(rag2:MYC) transgenic zebrafish also develop B-ALL [123,124,125,126]. While the *rag2* gene shows a specific expression in immature lymphoid cells, the zebrafish *rag2* promoter used in transgenics is active in both lymphoid and nonlymphoid cell populations including olfactory rosettes, sperm and mesenchymal progenitor cells [127,128,129]. Thus, the ectopic expression properties of the zebrafish *rag2* promoter in the mesenchymal cell compartments has been used to drive expression of *KRAS^G12D^* or a myristoylated constitutively active form of *Akt2* in order to generate transgenic models of rhabdomyosarcoma or liposarcoma, respectively [129,130]. Apart from *rag2*, a number of tissue-specific promoters have been used to drive oncogene expression into transgenic zebrafish and to induce cancer formation. These tissue-specific promoters include the myeloid-specific promoter *spi1* to induce AML [131,132,133], *mitfa* (melanocyte inducing transcription factor a) to induce melanoma [134,135,136,137], *cdh15* (M-cadherin) and *mylz2* (myosin light chain) to induce rhabdomyosarcoma [138], *ptf1a* (pancreas associated transcription factor 1a) or *myod* to induce exocrine or endocrine pancreatic carcinomas respectively [139,140], *fabp10a* (fatty acid binding protein 10a) to induce hepatocellular carcinoma or intrahepatic cholangiocarcinoma [141,142,143,144,145,146,147,148], *dbh* (dopamine β-hydroxylase) and *sox10* to induce brain tumors [149,150,151], *pomc* (proopiomelanocortin) to induce pituitary adenoma [151], *tg* (thyroglobulin) to induce papillary thyroid carcinoma [90] or *flck* (Fugu lymphocyte-specific protein tyrosine kinase) to induce testicular germ cell tumors [152] (Table A1).

Elizabeth Patton, Leonard Zon and colleagues (Children’s Hospital, Boston, MA, USA) used the melanocyte-specific promoter *mitfa* to drive the expression of the *BRAF^V600E^* oncogene [134]. The transgenic line develops patches of ectopic melanocytes, termed fish (f)-nevi, but not melanoma. This is similar to human pigmented nevi, a benign lesion that also carries *BRAF^V600E^* mutations at a high frequency [153]. However, when present in a *tp53^−/−^*genetic background, *BRAF^V600E^* expression leads to highly invasive and transplantable melanoma, highlighting a synergistic interaction between the BRAF and TP53 pathways in the development of melanoma. *NRAS^Q61K^* and *HRAS^G12V^* transgenic zebrafish have also been generated to model human melanoma [135,136]. While *NRAS^Q61K^*-dependent progression to melanoma requires concomitant *tp53* loss-of-function mutation, *HRAS^G12V^* targeted expression alone causes ectopic melanocyte formation during early embryogenesis, melanocyte hyperplasia, dysplasia, invasion of loose connective tissues and rapid progression to deeply invasive melanoma. As such, Tg(mifta:HRAS^G12V^) transgenics may serve as a model for familial atypical mole and melanoma (FAMM) syndrome. In addition to BRAF^G600E^ and NRAS^Q61K^, a number of oncogenes have been shown to synergize with tp53 mutations in transgenic zebrafish. For instance, wild-type transgenic zebrafish expressing the HBV X (HBx) antigen under the control of the liver-specific promoter *fabp10a* do not develop hepatocellular carcinoma (HCC) whereas *tp53^−/−^* transgenics do develop HCC [142,154]. In the same way, wild-type transgenic zebrafish expressing constitutively active Akt2 in mesenchymal progenitors develop well-differentiated liposarcoma (WDLPS) at an incidence rate of 8%, while this rate raises to 29% in a *tp53^−/−^* genetic background [130]. Similarly, loss of *nf1* function accelerates disease onset and increases the penetrance of MYCN-induced neuroblastoma in Tg(dbh:GFP-MYCN) transgenic zebrafish [155]. The transgenic line Tg(rag2:KRAS^G12D^) expresses *KRAS^G12D^* in undifferentiated muscle satellite cells and develop tumors that resemble embryonal rhabdomyosarcoma, the most common childhood soft-tissue tumor [129]. Co-injection of multiple transgenes driving the expression of fluorescent proteins under the control of promoters reflecting different stages of the muscle cell differentiation is an efficient method to label cell subpopulations. Applied to the Tg(rag2:KRAS^G12D^) model, this elegant strategy shows that rhabdomyosarcoma are composed of distinct cell populations that are dynamically reorganized during tumor growth [129,156].

Thus, the transgenic approach based on the expression of oncogenes under the control of tissue-specific promoters, eventually in combination with other markers or mutations, is a straightforward method to model specific cancer types in zebrafish. However, in some cases, the use of ubiquitous promoters has also proven efficacy in zebrafish cancer modeling. TEL-AML1 (also known as ETV6-RUNX1), generated by the t(12;21)(p13;q22) chromosomal translocation is the most common chimeric fusion gene in childhood cancer and is selectively associated with pre-B acute lymphoblastic leukemia (ALL). TEL-AML1 expression under the control of zebrafish *actin* or *Xenopus elongation factor 1α* ubiquitous promoters leads to lymphoid progenitor expansion that evolved in oligoclonal B-lineage ALL in about 3% of the transgenic zebrafish at the 8 to 12 months of age [157]. Interestingly, lymphoid-specific expression of TEL-AML1 from the *rag2* promoter failed to cause lymphoid hyperplasia, suggesting that the fusion protein acts prior to the committed lymphoid progenitor stage. Of note, in transgenic mouse models, expression of TEL-AML1 does not result in any hematological disorder, unless these mice are subjected to ENU or extremely low-frequency (ELF) magnetic fields exposure [158,159,160], highlighting the value of the TEL-AML1 transgenic zebrafish model.

### 6.2. The Gal4/UAS System

The Gal4/UAS system is a binary system derived from the yeast *Saccharomyces cerevisiae* and used to drive conditional transgene expression in zebrafish [161]. It consists of the transcriptional activator Gal4 that controls gene expression through the binding to its UAS (upstream activation sequence) recognition DNA motif. To achieve tissue-specific gene expression, Gal4 or a Gal4 derivative, is placed under the control of a tissue-specific promoter whereas a minimal promoter containing UAS sequences drives the expression of the gene of interest. The gene of interest whose expression strictly depends on Gal4 binding to the UAS will be exclusively expressed in the tissues where Gal4 is present (Figure 5b). The Gal4/UAS expression system has been implemented in various animal models including *Drosophila* [162], *Xenopus* [163] or mice [164], while Nico Scheer and José Campos-Ortega (Universität zu Köln, Cologne, Germany) were the first to apply this conditional expression technique to transgenic zebrafish in 1999 [165]. A key property of this system is the separate integration of the two components in a driver line expressing Gal4 and in an effector UAS line allowing the silent propagation of toxic or lethal genes and their conditional activation only in double-transgenic offspring from the crosses between driver and effector lines.

The versatility of the Gal4/UAS system has largely been applied to the generation of zebrafish cancer models. A transgenic Tg(UAS:GFP-HRAS^G12V^) line develops melanoma when crossed with lines expressing Gal4 under the *kita* enhancer [166], leukemia when crossed with lines expressing Gal4 under the *fli1* promoter [167], glioma when crossed with lines expressing Gal4 under the *zic4* enhancer [168] or chordoma when crossed with lines expressing Gal4 under the notochord-specific promoter *shhb* (also known as *twhh*, tiggywinkle hedgehog) promoter [169]. On the opposite, a same driver line can be used to assay for the tumorigenic activity of different oncogenes or diverse mutations within the same oncogene. For instance, while the *ptf1a* (pancreas associated transcription factor 1a) promoter is active in cerebellum, hindbrain and pancreas, Tg(ptf1a:Gal4; UAS:KRAS^G12V^) double-transgenics develop pancreatic tumors [170], whereas Tg(ptf1a:Gal4; UAS:AKT1^Myr^) present glioma [171]. In another study, the Steven Leach lab (Johns Hopkins Medical Institutions, Baltimore, MD, USA) used a Tg(ptf1a:Gal4) driver line to compare the ability of 12 different KRAS mutations to drive pancreatic tumorigenesis in vivo [172].

### 6.3. Cre-Mediated Recombination

The Cre-mediated recombination is another way to control transgene expression. Cre is a site-specific recombinase recognizing and inducing recombination at specific 34 bp target sites called loxP sites. Depending on the orientation and location of loxP sites on the DNA molecules, Cre-mediated recombination is able to induce different DNA rearrangement events. These events are the deletion of a DNA sequence located between two loxP sites in direct orientation, the inversion of a DNA sequence located between two loxP sites in an inverted orientation, the insertion of a circular DNA molecule containing a loxP site into another loxP site-containing DNA molecule or a chromosomal translocation between two loxP site-containing linear DNA molecules. In transgenic mouse cancer models, the Cre-loxP conditional system has been used to achieve tissue-specific expression of transgenes, often recapitulating aspects of the human disease [173,174]. In a classical setting, a target transgenic mouse line carrying a promoter and an oncogene separated by an intervening sequence that contains elements preventing expression of the oncogene and flanked by loxP sites in direct orientation is mated with an effector transgenic mouse line expressing the Cre recombinase. In the double-transgenic offspring, Cre-mediated recombination occurs at the loxP sites, the intervening STOP sequence is deleted, and the oncogene is then placed under the control of the promoter leading to tumor formation [173] (Figure 5c). The tight regulation of transgene expression by this binary system is essential since many oncogenes play crucial roles during development and continuous oncogene expression may cause embryonic lethality, abnormal development or infertility in the transgenics preventing the maintenance of the lines.

The first group to apply the Cre-loxP system to zebrafish cancer modeling was Langenau et al. [122] in order to bypass the early lethality of the Tg(rag2:GFP-Myc) T-ALL transgenic model. A Tg(rag2:loxP-DsRed-loxP-GFP-Myc) target transgenic line that expresses the red fluorescent protein DsRed under the control of the *rag2* promoter was generated. When Cre recombinase was introduced into the transgenic embryos via mRNA microinjection, the DsRed DNA part was excised allowing GFP-Myc expression and leukemia development. However, only partial Cre-mediated recombination occurred and only about 6% of the Cre-injected zebrafish developed T-ALL, whereas the incidence of T-ALL is 100% in the Tg(rag2:GFP-Myc) animals [121,122]. Using the same approach, the group of Jae-Hak Park (Seoul National University, Seoul, Korea) generated a Tg(nes:loxP-mCherry-loxP-GFP-KRAS^G12V^) transgenic line. The injection of Cre mRNAs places KRAS^G12V^ under the control of the *nestin* promoter leading to extensive apoptosis of neural progenitor cells followed by severe edema of the brain and early death of the injected transgenic zebrafish. However, overexpression of KRAS^G12V^ was not able to induce brain tumor development [175]. More recently, effector lines stably expressing Cre were generated to achieve tissue-specific expression of oncogenes as a rational alternative to Cre mRNA injection. Indeed, when an effector line expressing Cre under the control of the *ela3l* (elastase 3l) promoter was mated to a Tg(ubb:loxP-mCherry-loxP-GFP-KRAS^G12V^) transgenic line, mCherry was excised from the target transgene and GFP-KRAS^G12V^ expressed in the endocrine pancreas. As a result, 40% of the double-transgenic zebrafish develop pancreatic endocrine tumors by the age of 12 months [176].

### 6.4. Heat Shock Inducible Promoters

Genetic tools allowing temporal control of oncogene expression are essential in cancer modelling. In this regard, promoters of heat shock proteins can successfully be time-controlled in transgenic zebrafish [177,178]. In zebrafish, the *hsp70* (heat shock protein 70) promoter is widely used for raising ectopic and controlled expression of transgenes. This promoter consists in a 1.5 kbp genomic fragment containing contiguous 5-bp DNA consensus sequences known as heat shock elements (HSEs). These elements are binding sites for the heat shock transcription factors Hsf, which are repressed through the association with the Hsp90 protein complex under unstressed conditions. As protein unfolding increases with temperature, the chaperone protein Hsp90 is recruited from the Hsf-Hsp90 protein complexes, Hsf is released and free to bind to its DNA recognition sites to activate transcription [179]. The heat shock treatment of live zebrafish is usually carried out by soaking the embryos for 30 min in water at 38 °C.

The *hsp70* promoter has been used to generate zebrafish transgenic lines expressing various oncogenes in a controlled fashion (Figure 5d). For instance, the Marina Mione lab (Centre for Integrative Biology, University of Trento, Trento, Italy) generated a Tg(hsp70:GFP-HRAS^SG12V^) transgenic line expressing the *HRAS^G12V^* oncogene under the control of the *hsp70* heat-inducible promoter [180]. Twenty-four hours old transgenic embryos subjected to heat shock developed traits of a Costello syndrome-like phenotype associated with an increase in cellular hyperproliferation and senescence. However, no obvious cancer formation was described in this model. In contrast, expression of the oncogenic fusion proteins AML1-ETO (RUNX1-RUNX1T1) or BCR-ABL1 under the *hsp70* promoter is responsible for the development of myeloid leukemia-like lesions after the heat shock of the transgenic embryos [181,182], whereas expression of the human EWS-FLI1 (EWSR1-FLI1) fusion oncoprotein under the *hsp70* promoter induces tumors with histology strongly resembling that of human Ewing’s sarcoma in heat shock-treated zebrafish [183]. Li-Jing Shen, Fang-Yuan Chen and colleagues (Shanghai Jiao Tong University School of Medicine, Shanghai, China) used a bidirectional promoter containing 8 HSEs to drive the expression of the GFP marker on one side and of oncogenes, either MYCN or the fusion product RUNX1-EVI1 (RUNX1-MECOM), on the other DNA strand [184,185]. In both cases, heat shock treatments of the transgenic embryos led to an altered hematopoiesis and an increase in AML blasts, serving as models of myeloid malignancies.

One early application of heat shock promoters to zebrafish cancer models was their use to control Cre expression. The cross of a Tg(hsp70:Cre) transgenic line with the Tg(rag2:loxP-DsRed-loxP-GFP-Myc) T-ALL model leads to double-transgenics that develop leukemia in 81% of heat shocked larvae [186]. This indicates that expression of Cre from a stable controlled transgene is far more efficient than from Cre mRNA injection in embryos. However, due to leakiness of the *hsp70* promoter about 13% of the non-heat shocked double transgenics also develop leukemia. Nevertheless, Tg(hsp70:Cre) transgenic lines have been used to activate oncogene expression such as KRAS^G12D^ or NUP98-HOXA9, in double-transgenic zebrafish [187,188].

Thus, despite the leakiness of the hsp70 promoter and the limitation of the heat shock inducible approach to embryos, this transgenic strategy based on heat shock promoters successfully achieve temporal control of oncogene expression applied to cancer modelling in zebrafish.

### 6.5. Tetracycline Regulated Expression

The tetracycline (Tet) system comes from *Escherichia coli* where it controls the expression of genes involved in tetracycline resistance. This system relies on the tetracycline repressor (tetR) that reacts to tetracycline or to its more stable derivative doxycycline (Dox). Through the fusion of tetR to the transactivation domain of the transcription factor VP16, a Dox-controlled transactivator (tTA) has been generated [189]. In this system named Tet-OFF, tTA binds to tetracycline operator (tetO) sequences and activates transcription from a minimal promoter in absence of Dox, whereas Dox binding to tTA inhibits tTA-mediated transcriptional activation [190]. Activation of the Tet-OFF transcription then relies on the removal of Dox from organisms making this approach somehow inconvenient. From tTA, Manfred Gossen, Herman Bujard and colleagues (Universität Heidelberg, Heidelberg, Germany) developed a Dox-dependent transcriptional activator named rtTA [191]. In this Tet-ON binary system, the rtTA transactivator is inactive in absence of Dox, while upon Dox addition, rtTA binds to TetO-containing promoters and activate transcription (Figure 5e). The Tet-ON system has been applied to temporally regulate gene expression in a number of organisms, including *Drosophila* [192], mice [193] or zebrafish [194]. Conditional expression based on the Tet-ON system in transgenic zebrafish appears sometime leaky and whereas the induction by Dox is rapid and strong, inactivation of the transgene upon Dox removal is very slow. However, the rtTA system has been successfully used in transgenic zebrafish to model brain [195] and liver cancers [196]. In particular, Tet-ON-based strategies have been largely applied to hepatocellular carcinoma modelling by the group of Zhiyuan Gong (National University of Singapore, Singapore) [196,197,198,199,200,201]. In these studies, the oncogenes *xmrk*, *Myc* or *kras^G12V^* are expressed under the control of a tetO-containing minimal promoter, while rtTA expression is driven by the liver-specific promoter *fabp10a* [196,197,199]. In this manner, the spatial expression of the oncogene is achieved by the tissue-specific expression of rtTA and the temporal control relies on Dox supply. For instance, Tet-ON conditional expression of *xmrk*, a *Xiphophorus* hyperactive version of epidermal growth factor receptor, induces already after three weeks of Dox treatment, hepatocellular carcinoma leading to diminished growth and increased lethality in both juvenile and adult transgenic zebrafish. Moreover, induced liver tumors regressed rapidly upon inducer withdrawal, leading to complete rescue in about four weeks [196]. In another study, in order to investigate the relationship between Kras and RhoA, the Zhiyuan Gong lab used the Tet-ON strategy to express concomitantly in the same transgenics, the *kras^G12V^* oncogene on one side, and constitutively active (RhoA^G14V^) or dominant negative (RhoA^T19N^) versions of *rhoA* on the other side [199]. This approach not only shows that activation of RhoA inhibits the oncogenic effect of Kras^G12V^ but also demonstrates that the Tet-ON system could be applied to decipher signaling pathways involved in tumorigenesis.

### 6.6. Ligand-Binding Domain Fusion Proteins

Steroid hormone receptors are modular transcription factors organized into structurally and functionally defined domains [202,203]. Fusing the ligand-binding domain (LBD) onto other transcription factors or tyrosine kinases confers hormone-dependent activities on these proteins [204,205,206,207]. To activate the LBDs from estrogen receptor (ER), progesterone receptor (PR), glucocorticoid receptor (GR) and androgen receptor (AR), the most common hormone analogues used are 4-hydroxy-tamoxifen (4-OHT), mifepristone (RU486), dexamethasone and mibolerone, respectively (Figure 5f). The Thomas Look lab (Children’s Hospital, Harvard Medical School, Boston, MA, USA) used this conditional approach to control leukemia development in transgenic zebrafish [208]. A stable transgenic line Tg(rag2:MYC-ER^T2^), in which the zebrafish *rag2* promoter drives expression of human MYC oncogene fused to a modified LBD from the estrogen receptor (ER^T2^) that is posttranslationally induced by addition of 4-OHT but not by endogenous estrogens [209], has been generated. In this model, 4-OHT treatment induces MYC activation and disease development within 5 weeks of treatment, and withdrawal of 4-OHT results in T-ALL apoptosis and tumor regression. In contrast, untreated siblings do not develop blood abnormalities suggesting that the activity of the MYC-ER^T2^ fusion protein is tightly regulated without observable leakiness. Hence, using a tissue-specific promoter and a LBD fusion protein, two levels of regulation, spatial and temporal are combined in a single transgene to achieve oncogene activity control. In another work, Joji Nakayama, Zhiyuan Gong and colleagues described a transgenic zebrafish line expressing a Twist1a-ER^T2^ fusion driven by the liver-specific promoter *fapb10* together with the *xmrk* oncogene under the control of the Tet-ON system as a model for metastatic dissemination of liver cancer cells induced by both 4-OHT and Dox [210]. This possibility to activate simultaneously or sequentially different cancer genes confers a huge flexibility to the transgenic zebrafish cancer model.

LexPR results from the fusion of the DNA-binding domain of bacterial repressor protein LexA, a truncated LBD of the progesterone receptor and the activation domain from RELA (NF-αB/p65). The activation domain and the LexA DNA-binding motif make the LexPR a transcriptional activator recognizing LexO sequences and activating transcription from LexO-containing minimal promoters (LexOp). Furthermore, the use of progesterone receptor LBD containing a 42 amino acid C-terminal deletion renders the LBD unable to bind progesterone or other endogenous hormones but still able to bind mifepristone (RU486), a well characterized anti-progesterone compound [211]. When fused to other proteins, this truncated progesterone-binding domain confers RU486 responsiveness [212,213]. Then, the LexPR conditional system is a binary transcriptional system activating transcription from LexOp promoters in a mifepristone dependent fashion. Alexander Emelyanov and Serguei Parinov (National University of Singapore, Singapore) were the first to apply this system in transgenic zebrafish and to demonstrate its potential for oncogene activation [214]. One weakness of the system, also observed with other binary gene expression systems, is the variability in expression strength between larvae of the same clutch and between cells within the same larva. However, the LexPR system has been used in transgenic zebrafish to generate hepatocellular carcinoma models through the expression of LexPR under the control of the liver-specific promoter *fabp10a* and LexOp driving the expression of *kras^G12V^* or *tgfb1a* [215,216,217]. Similarly, when the LexOp Nras^Q61K^/Kras^G12V^ target oncogene is activated by LexPR under the control of the melanocyte-specific *mitfa* promoter or under the control of the intestine-specific *ifabp* (*fabp2*) promoter, double-transgenic zebrafish lines develop, in presence of mifepristone, melanoma or intestinal tumors, respectively [218,219]. In addition, the reversibility of the LexPR system allows the investigation of oncogene addiction of tumors in the transgenics. When Tg(fapb10:LexPR; LexOp:kras^G12V^) transgenics with apparent liver tumors, are transferred to mifepristone-free water, a regression of mifepristone-induced tumors is observed indicating that tumor maintenance requires continual kras^G12V^ expression [215]. Finally, the LexPR can be combined with other expression systems to develop more sophisticated zebrafish cancer models. For instance, the Tg(fabp10a:LexPR; LexOp:Cre; fabp10a:loxP-mCherry-loxP-GFP-kras^G12V^) transgenic line associate the LexPR system to Cre-mediated recombination [220]. These triple-transgenic zebrafish contain three different constructs obtained via crossbreeding of the driver/Cre-effector line with a fabp10a:loxP-mCherry-loxP-GFP-kras^G12V^ line. By applying RU486 to triple-transgenics, LexPR activator produced from the driver exclusively activates Cre expression in the liver, which subsequently removes DNA sequences coding for mCherry. After Cre-mediated recombination, the liver-specific expression of *kras^G12V^* is constitutively activated and the triple-transgenic zebrafish develop hepatocellular carcinoma.

Owing to the success of inducible Cre/loxP applications in mouse models [221,222], the chimeric Cre-LBD system has been also transferred into zebrafish to achieve spatial and temporal control of transgene expression [223] (Figure 5f). Additionally, the large number of CreER^T2^ expressing lines [224] offers a strong potential in terms of generation of zebrafish cancer models. However, only few applications of the conditional CreER^T2^ technology have been reported so far in the field of cancer modelling [225,226]. Kalasekar et al. [225] generated a model of hepatocellular carcinoma based on the CreER^T2^-mediated conditional expression of an activated version of β-catenin (Xla.Ctnnb1^S33A, S37A, T41A, S45A^, and hereafter-named Ctnnb1^ACT^). In this system, a Tg(fapb10a:CreER^T2^) line expressing Cre recombinase fused to the modified LBD of the estrogen receptor downstream of the hepatocyte-specific *fabp10a* promoter is used as a driver line, whereas a Tg(fapb10a:loxP-BFP-loxP-Ctnnb1^ACT^) transgenic line serves as a switch target line. Treatment of double-transgenic larvae with 4-OHT from 3 dpf to 6 dpf results in expression of activated β-catenin in most hepatocyte leading to hepatocellular carcinoma development in about 30% of the adult zebrafish at 3 months of age. However, untreated double-transgenics also develop hepatocellular carcinoma at a similar frequency. This observation is explained by a leakiness of the CreER^T2^ system. Indeed, using a fluorescent color switch Tg(ubi:loxP-GFP-loxP-mCherry) reporter line crossed with the CreER^T2^ driver line, the authors showed that at 6 dpf, 48% of the hepatocytes expressed mCherry upon 4-OHT treatment, but 6% of the hepatocytes are also mCherry^+^ in absence of treatment. Thus, a subset of larval hepatocytes expressing the activated β-catenin is sufficient to drive liver tumorigenesis. Furthermore, hepatocellular carcinoma penetrance was significantly lower in the CreER^T2^-based model (about 30%) than in the constitutive Tg(fabp10a:Ctnnb1^ACT^) model in which activated β-catenin is directly under the control of the *fabp10a* promoter (about 85%) [147]. One possible explanation for the difference in penetrance is that activated β-catenin is expressed slightly earlier in the constitutive Tg(fabp10a:Ctnnb1^ACT^) model than in the CreER^T2^-based model due to the time required to transcribe and translate CreER^T2^ and to excise the BFP/STOP cassette preventing the expression of the activated β-catenin [225]. Then, the potential leakiness and the possible reduced efficacy of the CreER^T2^ system imply the requirement for further improvements before its wider application to zebrafish cancer modelling. Finally, it worth noting that in contrast to the LexPR strategy, 4-OHT-mediated activation of the CreER^T2^ systems induces irreversible oncogene activation. This might not be a drawback in terms of cancer modelling since mutations are stably acquired during tumorigenesis [227,228], and in certain application such as drug screening, the maintenance of the mutation status in absence of the inducer could be an advantage.

### 6.7. Optogenetics

Optogenetics broadly refers to biological techniques involving light-gated proteins to control cellular behavior. It provides a precise tool to modulate spatially and temporally cellular activities [229,230,231]. Although optogenetics relies generally on light-sensitive protein domains, an optical control could also be applied to uncage the inducer of a protein activity. Zhiping Feng, Shimon Weiss, David Bensimon and co-workers (University of California at Los Angeles, Los Angeles, CA, USA; Ecole Normale Supérieure, Paris, France) reported an original approach to induce oncogene expression and tumor formation in transgenic zebrafish using optical control [232]. The strategy utilizes caged-cyclofen, a synthetically modified estrogen receptor inducer, which allows stringent light-dependent activation of proteins fused to the modified estrogen receptor LDB, ER^T2^. In presence of caged-cyclofen, these ER^T2^ fusion proteins are sequestered in the cytoplasm through their interaction with chaperones. Optical-induced uncaging of cyclofen releases the fusion proteins from their chaperone complexes, allowing them to translocate into the nucleus and activate transcription (e.g., Gal4) or induce recombination (e.g., Cre). Based on this principle, two light-inducible strategies for transient or constitutive *KRAS^G12V^* expression in transgenic zebrafish were implemented. The transient system combines the effector transgenic construct ubi:Gal4-ER^T2^ where the Gal4-ER^T2^ fusion is expressed under the control of the *ubiquitin* promoter, and the target transgene UAS:KRAS^G12V^-T2A-CFP. Photo-activation of caged-cyclofen allows Gal4-ER^T2^ to induce *KRAS^G12V^* expression. Once uncaged cyclofen diffuses, the expression of *KRAS^G12V^* is turned off and its mRNA and protein products are slowly degraded, resulting in a transient expression of the oncogene. Furthermore, the co-expression of the CFP fluorescent protein reports on the activity of the target transgene. The authors showed that transient activation of *KRAS^G12V^* through photo-activation of caged-cyclofen at 1 dpf does not result in tumor formation. In contrast, periodic *KRAS^G12V^* expression achieved by 1 day exposure to cyclofen every 5 days during 2 months, leads to tumor development in about 3% of the transgenic zebrafish within 12 months [232]. Constitutive *KRAS^G12V^* expression is obtained through the injection of the ubi:loxP-EosFP-loxP-KRAS^G12V^-T2A-mTFP target construct into transgenic Tg(ubi:CreER^T2^) zebrafish, subsequently exposed to caged-cyclofen prior photo-activation at 1 dpf. About 10% of treated embryos will then develop tumors before 1 year of age. Feng et al. [232] were also able to induce *KRAS^G12V^* expression at the single-cell level using two-photon uncaging, thus highlighting the potential of the approach although none of the fish developed a tumor in this condition.

### 6.8. Mosaic Somatic Transgenesis

The generation of stable transgenic zebrafish models of cancer is tedious and time-consuming, and even more difficult when the studies require the generation of multiple transgenic lines to activate oncogene expression or to investigate the cooperation of various genetic alterations. Mosaic transient (or somatic) transgenesis appears then as a powerful alternative strategy for rapid functional studies (Figure 4b) [233]. One or several transgenes are injected into one-cell stage wild-type or transgenic embryos, and randomly integrates into the genome of the F0 mosaic zebrafish [234]. Co-injection of different transgenes in the embryo leads to their co-integration into given cells allowing the co-expression of the transgenes in these cells [235]. Moreover, given that cancer generally results from somatic sequential mutations rather than multiple germline genetic alterations, the mosaic transient transgenesis approach has also a rational basis. The Leonard Zon Lab (Children’s Hospital, Boston, MA, USA) has largely demonstrated that the simplicity of the technique allows rapid and robust melanoma modelling by combining mosaic somatic expression and/or inactivation of different oncogenes and tumor-suppressor genes in zebrafish [83,236,237]. This approach has also been applied to demonstrate the oncogenic role of long non-coding (lnc) RNAs such as *THOR* [238].

Oncogene expression through transgenesis via the injection of transgenes into one-cell stage embryos implies that these oncogenes will be expressed from early embryogenesis, with potentially deleterious effects during development. To bypass this possible limitation, Richard White and colleagues (Memorial Sloan Kettering Cancer Center, New York City, NY, USA) implemented a method called TEAZ (Transgene Electroporation in Adult Zebrafish) to deliver transgenes driving oncogene as well as CRISPR/Cas9 components expression, directly into somatic tissues of adult zebrafish using electroporation [239]. One interesting outcome of this approach is that it is possible to track over the time the spreading of the tumor from its origin, at the electroporation point.

Then, the continuous progress in the improvement of conditional transgenesis approaches in zebrafish has generated an immense toolbox for controlling oncogene and tumor-suppressor gene activities in specific cells and a given time. This set of tools offers a variety of modelling possibilities in the field of cancer research.

## 7. Conclusions

Over the past two decades, the progresses accomplished in the implementation of sophisticated genetic and reverse genetic tools allowed the emergence of a remarkable diversity of genetically engineered zebrafish cancer models. The precision of the reverse genetic toolbox enables the generation of zebrafish lines carrying genetic modifications that are identical to the alterations found in patients. Transcriptomic and oncogenomic comparisons between human and zebrafish tumors reveal common molecular signatures and potentially lead to the discovery of novel genes involved in tumorigenesis [29,81,90,129,135,240,241]. The possibility to associate several genetic alterations in zebrafish allows combinatorial genetic modelling to recapitulate the genetic alterations found in humans and to decipher the cross-talk between different pathways involved in tumor initiation or progression [83,149,199]. Thus, the zebrafish contributes novel insights in tumor biology and provides suited cancer models that can ultimately being used to identify anti-cancer drugs [242,243].

## Figures and Tables

**Table 1:** Zebrafish cancers induced by chemical exposure.

Chemicals	Cancer Types	References
NDMA	Hepatocarinoma	[26]
DEN	Liver and pancreas carcinomas	[27]
DMBA	Liver, gill, pancreas, gastrointestinal, pancreas, testis, cartilage, muscle, blood vessel, connective and lymphoid tissues and neural tumors	[28,29]
DBP	Hepatocarcinoma	[29]
MNNG	Liver, gill, pancreas, gastrointestinal, pancreas, testis, kidney, muscle tumors and hemangio(sarco)mas	[30]
ENU	Epidermal papillomas	[31]

DBP, dibenzo(a,l)pyrene; DEN, diethylnitrosamine (N-nitrosodimethylamine); DMBA, 7,12-dimethylbenz[*a*]anthracene; ENU, N-ethyl-N-nitrosourea; MNNG, N-methyl-N’-nitro-N-nitrosoguanidine; NDMA, N-nitrosodimethylamine.

**Table 2:** Cancer genes identified in zebrafish mutagenesis screens.

Mutagenesis	Mutated Genes	Cancer Phenotypes	References
ENU	*dnaaf1* (*lrcc50*)	Seminoma (incidence: 90% after 2 years of age)	[40]
ENU	*bmpr1bb* (*alk6b*)	Testicular germ cell tumor	[41]
Retroviral insertion	Ribosomal proteins (*rps8, rps15a, rpl7, rpl35, rpl36, rpl36a, rpl13, rpl23a, rps7, rps18, rps29*)	MPNST (incidence: 14% to 100% at 2 years of age)	[43]
Retroviral insertion	*nf2a*	MPNST	[43]
Retroviral insertion	*fgf8* (overexpression)	Neuroblastoma (incidence: 25–50% at 2 years of age)	[44]
ENU	*mybl2b (bmyb)* (Mutant crash&burn)	Altered cell proliferation and genome instability Increased MNNG-induced cancer susceptibility	[45]
ENU	*espl1* (*separase*) (Mutant cease&desist)	High levels of polyploidy and aneuploidy. Increased MNNG-induced cancer susceptibility	[46]
ENU	*sufu* (Mutant dre) *hhip* (Mutant uki) *ptch1* (Mutant lep)	Activation of the Hedgehog signaling pathway	[47]
ENU	*tp53*	Deficiency in radiation-induced apoptosis Sarcomas (100% penetrance in adults)	[50]
ENU	*llgl2* (*lgl2*) (Mutant pen)	Epidermal cell tumor	[51,52]
ENU	*myh11* (Mutant mlt)	Epithelial invasion and cystic expansion of the intestine	[53]

ENU, N-ethyl-N-nitrosourea; MNNG, N-methyl-N’-nitro-N-nitrosoguanidine; MPNST, malignant peripheral nerve sheath tumors.

**Table 3:** Zebrafish mutants generated through the TILLING approach.

Gene	Cancer Types	References
*tp53*	MPNSTs (Incidence: 28% at 8.5 months of age)	[60]
*ptena, ptenb*	Ocular hemangiosarcomas (Incidence: 10.2% at 12 months of age)	[61,62]
*apc*	Intestinal, hepatic and pancreatic proliferative neoplasia (Incidence: 29% at 15 months of age)	[63]
*mlh1, msh2, msh6*	Primarily neurofibromas in the eye and abdomen. (Incidence: about 33% at 18 months of age)	[64]
*brca2*	Testicular Neoplasia (Incidence: 31% at 10–16 months of age)	[65]

MPNST, malignant peripheral nerve sheath tumor.

**Table 4:** Cancer genes targeted by programmable site-specific endonucleases in zebrafish.

Nuclease	Mutated Genes	Cancer Types	References
ZFN	*nf1a, nf1b*	High grade gliomas and MPNSTs in the *tp53^−/−^*genetic background	[76]
ZFN	*tet2*	Generalized MDS at 24 months of age	[77]
TALEN	*rb1*	Primitive neuroectodermal tumors (Incidence: 11–33% at 3.5 months of age in F0 mosaics)	[78,79,80]
TALEN	*cdkn2a/b*	MPNSTs in F0 *tp53^−/−^*mutants (Incidence: 39% at 35 weeks of age)	[79]
TALEN	*tp53^del/del^*	Angiosarcoma, MPNSTs, germ cell tumors (Incidence: 37% at 12 months of age)	[81]
TALEN CRISPR	*irx1a* *irx1b*	Intestinal hyperplasia and testicular, ovarian, renal and bile duct tumors	[82]
CRISPR	*spred1, tp53, ptena/b, cdkn2a*	Melanocyte-specific tumor suppressor gene inactivation promotes KIT-, BRAF- and NRAS-driven melanoma formation	[83]
CRISPR	*ptch1*	T-cell-specific inactivation promotes NOTCH1^ICD^-driven T-ALL development	[84]
CRISPR	*atrx*	Hemizygous loss of *atrx* promotes the development of epithelioid sarcoma, angiosarcoma and pleomorphic sarcoma in *tp53*/*nf1*-deficient mutants	[85]
CRISPR	*suz12a, suz12b*	Loss of *suz12* function accelerates the onset of MPNST and expands the spectrum of tumor types in *tp53*/*nf1*-deficient mutants	[86]
CRISPR	*arid1aa, arid1ab*	*arid1aa* or *arid1ab* deficiency increases the penetrance of MYCN-induced neuroblastoma in Tg(dbh:EGFP-MYCN) transgenics	[87]
CRISPR	*lats2*	PNST in F0 mosaics	[88]
CRISPR	*dact2*	Tumor formation in the digestive system (active proliferation and hyperplasia in the pancreatic duct, the intrahepatic bile duct, the intestinal epithelium, pancreas and liver)	[89]
CRISPR	*twist3*	Loss of *twist3* prevents BRAF^V600E^-mediated tumorigenesis in thyroid	[90]

MDS, myelodysplastic syndrome; MPNST, malignant peripheral nerve sheath tumor; PNST, peripheral nerve sheath tumor; T-ALL, T-cell acute lymphoblastic leukemia.

## Appendix A

**Table A1:** Transgenic zebrafish lines developing cancers.

Transgenic Lines	Cancer Types	References
Tg(rag2: GFP-Myc)	T-ALL	[121]
Tg(rag2:loxP-DsRed-loxP-GFP-Myc); mRNA-Cre		[122]
Tg(rag2:loxP-DsRed-loxP-GFP-Myc; hsp70:Cre)		[186]
Tg(rag2:MYC-ER^T2^)		[208]
Tg(rag2:NOTCH1^ICN^-GFP)		[244]
Tg(rag2:GFP-Myc; rag2:GIMAP5; rag2:GIMAP7)		[245]
Tg(rag2:GFP-bcl2)	T-LBL	[246]
Tg(rag2:GFP-Myc; rag2:GFP-bcl2)		
Tg(act/xEF1:TEL-AML1)/[ETV6-RUNX1]	B-ALL	[157]
Tg(rag2: GFP-Myc)		[123]
Tg(rag2:MYC)		[124,125]
Tg(spi1:MYST3-NCOA2-GFP) [MOZ-TIF2]	AML	[131]
Tg(hsp70:AML1-ETO)		[181]
Tg(MYCN:HSE:GFP)		[184]
Tg(RUNX1-EVI1:HSE:GFP)		[185]
Tg(spi1:tel-jak2a)/Tg(CMV:tel-jak2a)		[247,248]
Tg(spi1:loxP-GFP-loxP-NUP98-HOXA9; hsp70:Cre)		[188]
Tg(fli1:Gal4FF; UAS:GFP-HRAS^G12V^)		[167]
Tg(hsp70:p210^BCR/ABL1^)		[182]
Tg(spi1:FLT3^ITD^-2A-GFP)		[132]
Tg(spi1:SOX4-GFP)		[133]
Tg(mitfa:BRAF^V600E^); tp53^−/−^	Melanoma	[134]
Tg(mitfa:BRAF^V600E^); mitfa^vc7/vc7^		[249]
Tg(mitfa:GFP-NRAS^Q61K^); tp53^−/−^		[135]
Tg(mitfa:mitfa; mitfa:NRAS^Q61K^); Casper		[237]
Tg(mitfa:HRAS^G12V^; mitfa:GFP)		[136]
Tg(UAS:GFP-HRAS^G12V^; mitfa/kita:Gal4-VP16)		[166,250]
Tg(mitfa:GNAQ^Q209P^); tp53^−/−^		[137]
Tg(kita/mitfa:LexPR-Cerulean; lexOp:RFP-Kras^G12V^/Hras^G12V^/Nras^Q61K^)		[218]
Tg(mitfa:BRAF^V600E^); tp53^−/−^; prdm1a^+/−^		[251]
Tg(rag2:KRAS^G12D^)	Rhabdomyosarcoma	[129]
Tg(cdh15:KRAS^G12D^), Tg(mylz2:KRAS^G12D^)		[138]
Tg(ptf1a:GFP-KRAS^G12V^)	Pancreas tumors	[139]
Tg(ptf1a:GAL4-VP16; UAS:KRAS^mut^)		[170,172]
Tg(myod:MYCN)		[140]
Tg(ptf1a:CreER^T2^; ubb:loxP-STOP-loxP-Gal4; UAS:KRAS^G12V^)		[226]
Tg(ela3I:Cre; ubb:loxP-STOP-loxP-KRAS^G12D^)		[176]
Tg(act:loxP-EGFP-loxP-KRAS^G12D^; hsp70:Cre)	Various	[187]
Tg(act: EWS-FLI1)/Tg(hsp70: EWS-FLI1) (tp53*^–/–^*)	Ewing sarcoma	[183]
Tg(rag2:myr-Akt2)	Liposarcoma	[130]
Tg(krt4:myr-AKT1)		[252]
Tg(fabp10a:GFP-kras^G12V^)	Liver tumors	[141]
Tg(lexA:GFP-kras^G12V^; fapb10a:LexPR)		[215]
Tg(TetO:Myc; fapb10a:rtTA)		[197,198]
Tg(TetO:xmrk; fapb10a:rtTA)		[196,198]
Tg(TetO:xmrk; fapb10a:rtTA; fabp10a:mCherry-T2A-twist1a-ER^T2^)		[210]
Tg(fabp10a:HBx-RFP); tp53^−/−^/Tg(fabp10a:src); tp53^−/−^		[142,201]
Tg(fabp10a:UHRF1-GFP)		[143]
Tg(fabp10a:rtTA2s-M2; TRE2:EGFP-kras^G12V^)		[199,200,201]
Tg(fabp10a:rtTA2s-M2; TRE2:EGFP-kras^G12V^; TRE2:EGFP-rhoA^T19N^)		[199]
Tg(fabp10a:nras^Q61K^)		[144]
Tg(fabp10a:RPIA)		[145]
Tg(fabp10a:LexPR; LexOp:Cre; fabp10a:loxP-mCherry-loxP-EGFP-kras^G12V^)		[220]
Tg(fabp10a:LexPR-T2a-mcherry; LexOp:tgfb1a)		[216,217]
Tg(fabp10a: AURKA^V352I^; myl7:EGFP)		[146]
Tg(fabp10a: Xla.Ctnnb1^S33A, S37A, T41A, S45A^)		[147]
Tg(fabp10a:CreER^T2^; fapb10a:loxP-BFP-loxP-Xla.Ctnnb1^S33A, S37A, T41A, S45A^)		[225]
Tg(fapb10a:edn1)		[148]
Tg(ifapb:LexPR; LexOp:EGFP-kras^G12V^)	Intestinal tumors	[219]
Tg(UAS:GFP-HRAS^G12V^; twhh/mü4465:Gal4)	Chordoma	[169]
Tg(UAS:myr-AKT1; ptf1a:Gal4-VP16)	Brain tumors	[171]
Tg(UAS:GFP-RAC1^G12V^; ptf1a:Gal4-VP16)		[171]
Tg(UAS:smoa1-GFP; krt5:Gal4-VP16)		[253]
Tg(UAS:mCherry-KRAS^G12V^; krt5/gfap:Gal4-VP16)		[195]
Tg(TRE:mCherry-KRAS^G12V^; krt5/gfap:rtTa)		[195]
Tg(UAS:GFP-HRAS^G12V^; zic4:Gal4-VP16)		[168]
Tg(dbh:GFP-MYCN), Tg(dbh:ALK^F1174L^)		[149]
Tg(dbh:EGFP-MYCN; dbh:LMO1)		[254]
Tg(dbh:EGFP-MYCN); nf1a^−/−^		[155]
Tg(sox10:mCherry-NRAS/NRAS^Q61R^/NRAS^S17N^); p53^−/−^		[150]
Tg(dbh:MYC)/Tg(dbh:MYCN)		[151]
Tg(dbh:GFP-MYCN; dbh:LIN28B)		[255]
Tg(actb2:KIT^D816V^-GFP)	Mastocytosis	[256]
Tg(pomc:pttg; POMC:GFP)	Pituitary adenoma	[257]
Tg(tg:BRAF^V600E^; tg:TdTomato)	Papillary thyroid carcinoma	[90]
Tg(flck:Tag)/Tg(flck:scl)/Tg(flck:scl; flck:lmo1)	Testicular germ cell tumors	[152]
Tg(ubi:loxP-EosFP-loxP-KRAS^G12V^-T2A-mTFP; ubi:CreER^T2^)	Abdominal tumors	[232]
Tg(UAS:KRAS^G12V^-T2A-CFP; ubi:Gal4-ER^T2^)		
